# Cardiac fibroblasts, fibrosis and extracellular matrix remodeling in heart disease

**DOI:** 10.1186/1755-1536-5-15

**Published:** 2012-09-03

**Authors:** Dong Fan, Abhijit Takawale, Jiwon Lee, Zamaneh Kassiri

**Affiliations:** 1 Department of Physiology, University of Alberta, Edmonton, AB T6G 2S2, Canada; 2 Mazankowski Alberta Heart Institute/Cardiovascular Research Center, University of Alberta, Edmonton, AB T6G 2S2, Canada

**Keywords:** Cardiac fibroblast, Matrix metalloproteinases, Tissue inhibitor of metalloproteinases, Extracellular matrix remodeling, Heart disease

## Abstract

Fibroblasts comprise the largest cell population in the myocardium. In heart disease, the number of fibroblasts is increased either by replication of the resident myocardial fibroblasts, migration and transformation of circulating bone marrow cells, or by transformation of endothelial/epithelial cells into fibroblasts and myofibroblasts. The primary function of fibroblasts is to produce structural proteins that comprise the extracellular matrix (ECM). This can be a constructive process; however, hyperactivity of cardiac fibroblasts can result in excess production and deposition of ECM proteins in the myocardium, known as fibrosis, with adverse effects on cardiac structure and function. In addition to being the primary source of ECM proteins, fibroblasts produce a number of cytokines, peptides, and enzymes among which matrix metalloproteinases (MMPs) and their inhibitors, tissue inhibitor of metalloproteinases (TIMPs), directly impact the ECM turnover and homeostasis. Function of fibroblasts can also in turn be regulated by MMPs and TIMPs. In this review article, we will focus on the function of cardiac fibroblasts in the context of ECM formation, homeostasis and remodeling in the heart. We will discuss the origins and multiple roles of cardiac fibroblasts in myocardial remodeling in different types of heart disease in patients and in animal models. We will further provide an overview of what we have learned from experimental animal models and genetically modified mice with altered expression of ECM regulatory proteins, MMPs and TIMPs.

## Review

### Cardiac fibroblasts

Myocardium is comprised of a number of cell types, cardiomyocytes, cardiofibroblasts, endothelial cells and smooth muscle cells. Cardiac fibroblasts (CFBs) have the highest cell population in the myocardium, accounting for about two-thirds of the cells, while cardiomyocytes constitute about two-thirds of the myocardial tissue volume [1], although this ratio may vary in different species [2]. A number of excellent reviews have discussed the contribution of the contractile proteins and the molecules involved in intracellular calcium handing in cardiomyocytes in cardiac pathologies [3-5]. In this review, we will provide an overview of the literature on the role of CFBs in the context of extracellular matrix (ECM) remodeling and its contribution to development and progression of heart disease. Fibroblasts (FBs) are cells of mesenchymal origin and are present in every tissue in the body [2,6]. Morphologically, FBs are flat and spindle-shaped with multiple projecting processes. In the myocardium, CFBs are unique among other cell types in that they lack a basement membrane. Although historically FBs were considered a homogeneous cell population, it has become increasingly clear that FBs from different tissues have different properties and functions [2,7]. In this review we will focus our discussion on CFBs, although some of the discussed properties and functions could also apply to FBs from other tissue sources.

A number of cell surface markers have been identified for FBs and CFBs, but over time their specificity to these cells has been challenged. Vimentin, a protein that is present in the intermediate filaments of FBs, has been the most widely used FB marker – and although it is also expressed in other cell types such as endothelial cells [8] and myoepithelial cells [9], due to morphological differences among these cell types, vimentin remains a reliable marker for identifying FBs [10]. Discoidin domain receptor (DDR) 2 was discovered as a specific marker for CFBs [1,2,11]. DDR1 and DDR2 are collagen receptors [12,13], a family of protein tyrosine kinases involved in a variety of cellular functions such as growth, migration and differentiation [14]. DDR1 is expressed mainly in epithelial cells, whereas DDR2 is expressed in mesenchymal cells [15]. DDR2 was detected in rat and mouse heart [16], and has been considered to be more specific than vimentin for CFBs since it is not expressed in cardiomyocytes or cardiac endothelial cells [11]; however, it is also expressed on specific bone-marrow-derived cells, fibrocytes [17], leukocytes, vascular smooth muscle cells [18,19], and corneal epithelial and endothelial cells [20]. Another FB marker is fibroblast-specific protein 1, a filament-associated calcium-binding protein in FBs [21]; however, fibroblast-specific protein 1 has also been found to be expressed in leukocytes and a number of cancer cells [22].

### Myofibroblasts

In response to appropriate stimuli, most commonly myocardial injury, CFBs can differentiate into myofibroblasts (myoFBs), which are more mobile and contractile with a greater synthetic ability to produce ECM proteins [23]. MyoFBs, originally identified and named by Gabbiani in 1971 [24], are not found in healthy myocardium and only appear following cardiac injury [25]. Similar to CFBs, cardiac myoFBs are nonexcitable cells, but express a number of smooth muscle cell markers that are not typically expressed in quiescent CFBs, such as alpha smooth muscle actin (αSMA) [26], smooth muscle myosin heavy chain, vinculin, paxillin, and tensin [27]. The internal microfilmanents in the myoFBs are connected to the extracellular fibronectin domains via specialized adhesion complexes called fibronexus. This allows the myoFBs to exert a contractile force on the surrounding ECM [28]. MyoFBs are highly responsive to chemokines released at the site of injury. This is the main mechanism that mediates migration of FBs to the site of injury. In addition, myoFBs themselves produce and secrete a number of cytokines (for example, IL-1α, IL-1β, IL-6, IL-10 and TNFα), which in turn help to maintain the inflammatory response to injury [25].

Cardiac injury triggers CFBs to be differentiated to myoFBs, which have a stronger ability to produce ECM proteins. MyoFB have been demonstrated to play a key role in reparative fibrosis in the infarcted heart [29], and to be associated with hypertrophic fibrotic scars in various injury models. Differentiation from FB to myoFB is promoted by transforming growth factor beta (TGFβ), cytokines, the ECM, and other growth factors [30,31]. TGFβ induces the transdifferentiation of CFBs into myoFBs and increases collagen expression [23], whereas IL-1β inhibits differentiation of CFBs by preventing the expression of αSMA and other contractile proteins in these cells [25]. *In vitro*, αSMA expression levels in cultured CFBs are increased by passaging, and after the third passage CFBs are believed to become myoFBs [25] with elevated TGFβ expression such that stimulation with exogenous TGFβ could not further increase collagen production in these cells [23]. Transformation of CFBs to myoFBs shifts the balance in ECM turnover, increasing synthesis and accumulation of fibrotic depositions that can replace the myocytes and/or interrupt the myocyte–myocyte interactions in the myocardium leading to overall impairment of cardiac function.

### Origins of cardiac fibroblasts

CFBs are derived from mesenchymal cells. During heart development, epicardial cells formed by migration of proepicardial cells over the embryonic heart undergo epithelial-to-mesenchymal transformation and subsequently differentiate into FBs [32]. This transition is induced primarily by periostin [33] and TGFβ [34]. However, epicardial cells do not constitute all of the FBs in the heart. In principle, they only contribute to the FBs in the cardiac interstitium [35] and fibrous annulus [36,37]. The annulus is an electrically inert structure that forms the isolating barrier between the atrial and ventricular tissues necessary for normal sequential activation of the heart. The FBs in the atrioventricular valve leaflets are primarily derived from the endocardium [38]. After the completion of embryonic development, the epicardium-derived and endocardium-derived cells become quiescent.

**Figure 1:**
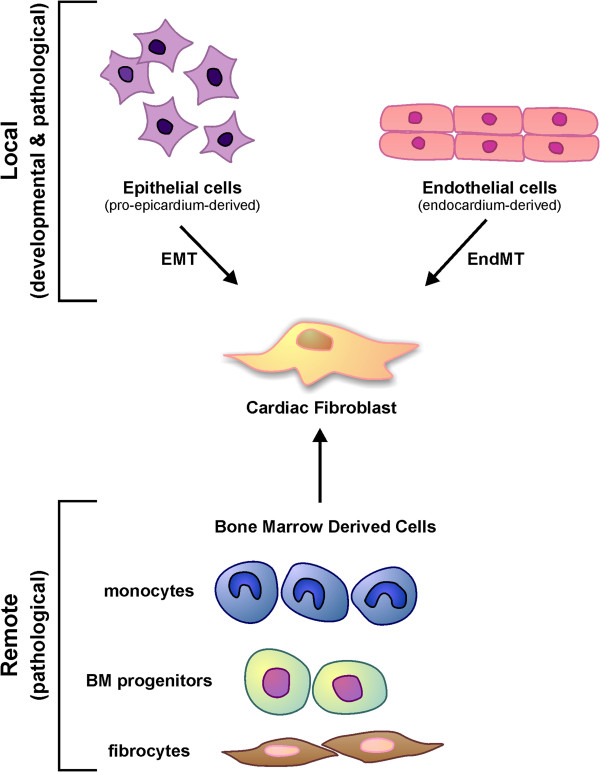
**Origin of cardiac fibroblasts during development and disease.** During development, epicardium-derived cells undergo epithelial–mesenchymal transformation (EMT), while endothelial cells (from the endocardium) can undergo endothelial–mesenchymal (EndMT) and transform to cardiac fibroblasts. Following myocardial injury, bone marrow (BM)-derived cells (monocytes, BM progenitors and fibrocytes) can be recruited to the site of injury and transformed to cardiac fibroblasts. This can occur in addition to EMT and/or EndMT.

Injury to the heart can trigger amplification of resident CFBs, transformation of endothelial or epithelial cells to FBs, or recruitment of hematopoietic cells originating from the bone marrow to the site of injury and their transformation into CFBs and myoFBs (Figure 1). Myocardial fibrosis in response to cardiac pressure overload is a characteristic feature of this disease and has been reported to result from proliferation of resident CFBs [39] as well as transformation of endothelial cells to mesenchymal cells leading to generation of CFBs and myoFBs [40,41]. Endothelial-to-mesenchymal transformation can be induced by TGFβ in a Smad-dependent fashion during cardiac fibrosis, while bone morphogenic protein 7 blocks this process and could serve as an anti-fibrotic factor [40]. A very recent study has reported that suppression of receptor kinase Tie-1, but not Tie-2, promotes endothelial-to-mesenchymal transformation in human endothelial cells [42]. In addition, Notch-mediated epithelial-to-mesenchymal transformation has also been reported to lead to CFB proliferation in myocardial infarction (MI) as well as in aortic constriction [43].

Bone marrow-derived cells and circulating blood cells such as monocytes and fibrocytes are another source of CFBs in response to injury [6]. Monocytes have been proposed as a potential source of CFBs in pathological fibrosis in ischemia–reperfusion injury through elevated monocyte chemoattractant protein-1, which promoted the uptake of hematologic monocytes [44], and MI [45,46]. Fibrocytes, circulating FB progenitor cells, originate from the hematopoietic stem cells in the bone marrow and display phenotypic similarities to other leukocytes such as CD14 expressed by monocytes [47]. Following MI, bone-marrow-derived cells were found to constitute a large number of CFBs and myoFBs in the infarct area contributing to infarct formation [45,48]. In addition, bone-marrow-derived cells were found to constitute more than 60% of the CFBs and myoFBs in an experimental autoimmune myocarditis model [49], and about 30% of CFBs and myoFBs in cardiac pressure overload [40]. CCR2, a chemokine receptor that is expressed on bone marrow cells, has been shown to be critical in recruitment of bone marrow cells to the heart during disease since CCR2 deficiency prevented angiotensin-II-induced accumulation of bone-marrow-derived FB precursors (fibrocytes) in the myocardium and cardiac fibrosis [50]. Perivascular cells, such as pericytes, have been shown to differentiate into collagen-producing FBs in the kidney [51] and in the retina *in vitro*[ 52], but the contribution of these cells in formation of CFBs has not yet been determined [6]. In summary, the origin of CFBs during development is different from that during disease, which could explain the different functions and properties of CFBs during development, health and disease.

### Functions of cardiac fibroblasts

**Figure 2:**
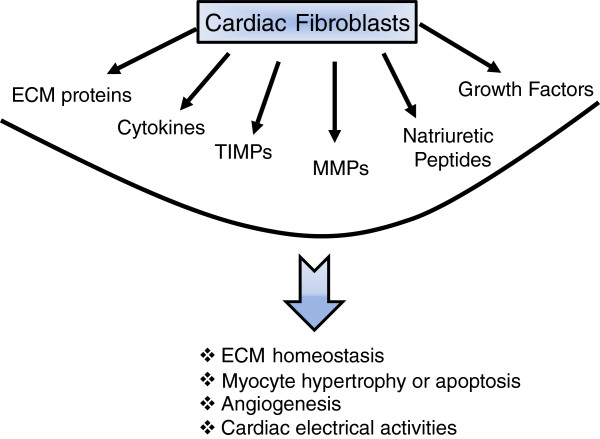
**Pluripotent cardiac fibroblasts impact different aspects of cardiac structure and function.** Cardiac fibroblasts can produce a number of active peptides (for example, cytokines, growth factors, peptides), extracellular matrix (ECM) proteins (collagens, elastin, fibronectin, and so forth), and ECM-regulatory proteins, matrix metalloproteinases (MMPs) and tissue inhibitors of matrix metalloproteinases (TIMPs). As such, cardiac fibroblasts can impact molecular and cellular events that collectively determine cardiac structure and function.

CFBs are involved in many aspects of cardiac functions, such as homeostasis and remodeling of the cardiac ECM, cell–cell communication with cardiomyocytes, electrical activity, production of growth factors and cytokines, and intercellular signaling with other CFBs, endothelial or smooth muscle cells that can impact cellular events such as angiogenesis, cell proliferation, cardiomyocyte hypertrophy or apoptosis (Figure 2). FBs can also be reprogrammed into different cell types, such as pluripotent stem cells [53], myoblasts [54], neurons [55]. Recently, it has been reported that FBs can be reprogramed to contracting cardiac-like myocytes cells by expressing developmental transcription factors, MEF2, HAND2, GATA4 and TBX5 [56,57], or by treatment with a combination of miRNAs (miRNAs 1, 133, 208 and 499) [58]. As such, CFBs are critical in maintaining normal cardiac structure, function, biochemical and electrical features of the heart, and CFBs also play a key role during pathological remodeling of the heart. CFBs are conductors with a high membrane resistance [59] and electrically separate the atria and the ventricle, by forming the fibrotic annulus, to ensure proper contraction of the heart [36]. CFBs are connected with cardiomyocytes via gap junctions, particularly connexins (Cx40, Cx43, and Cx45), which is essential in maintaining an optimal electrical conduction in the heart [10,60]. Another major function of CFBs is to synthesize a variety of bioactive molecules and secrete them into the myocardial interstitium. These molecules include cytokines (TNFα, interleukins and TGFβ), active peptides (angiotensin II, endothelin 1) and growth factors [61], which function in the myocardium in autocrine and/or paracrine fashions. CFBs are the key cell type responsible for ECM homeostasis in health and its remodeling in heart disease. CFBs synthesize the ECM proteins while also producing the enzymes that degrade these proteins, and inhibitors of these enzymes. In this review, we will focus on the function of CFBs in the context of ECM formation, homeostasis and remodeling in different types of heart disease.

### Cardiac fibroblasts are critical in extracellular matrix homeostasis

One of the primary functions of CFBs is maintaining the integrity of the cardiac ECM, a network structure that in addition to providing structural and functional integrity to the heart, also contains a number of cytokines and growth factors that can impact cardiac function and the fate of cardiac cells. Cardiac ECM is critical in mediating the mechanical connection among the cardiomyocytes, CFBs and the blood vessels within the myocardium. The ECM also transmits extracellular mechanical signals to the cardiomyocytes. The ECM is mainly comprised of fibrillar collagen types I and III, as well as less abundant collagen types IV, V and VI. The ECM also includes fibronectin, laminin, elastin and fibrillin, proteoglycans and glycoproteins. CFBs are the primary source of all of these ECM proteins [62], which can be induced by a number of growth factors such as platelet-derived growth factor, basic fibroblast growth factor and TGFβ during development and disease [63].

In addition to producing ECM proteins, CFBs also produce ECM-regulatory proteins – matrix metalloproteinases (MMPs), which can degrade ECM proteins – and their inhibitors, tissue inhibitors of metalloproteinases (TIMPs). A well-controlled balance between the function of MMPs and TIMPs is critical in maintaining ECM homeostasis [64]. MMPs are the predominant proteases responsible for degradation of the ECM proteins. MMPs are Zn^2+^-activated proteases that are synthesized as inactive zymogens (pro-MMPs), and can be activated by removal of an amino-terminal propeptide domain and exposure of the catalytic domain. Among the 26 MMPs cloned and characterized in vertebrates, the MMPs so far identified to be involved in myocardial remodeling are as follows: MMP1, MMP3, MMP8, MMP13, MMP2, MMP9, MMP12, MMP28 and the membrane-type MMPs (MT1-MMP/MMP14) [65-69], although the role of higher MMPs in the cardiovascular system is less well explored. MMP1 degrades collagen types I, II and III and the basement membrane proteins, MMP12 targets elastin, MMP8 and MMP13 can process collagen types I, II and III, while MT1-MMP can cleave a number of ECM proteins including fibronectin, laminin-1 and fibrillar collagen type I [70-73]. Although classically known as gelatinases, MMP2 and MMP9 also process a number of collagens, including collagen types I, IV and V, while MMP2 additionally cleaves collagen type III [74]. Rodents lack the MMP1 gene but express MMP1a (mColA) and MMP1b (mColB) genes [75], primarily in the reproductive organs but not in the heart [76]. The proteolytic activity of MMPs is kept in check by TIMPs, the predominant inhibitors of MMPs in the myocardium [64]. Four TIMPs have so far been cloned [77], among which TIMP2, TIMP3 and TIMP4 are expressed in the healthy heart, whereas TIMP1 is expressed at low levels in the healthy heart but its levels rise in diseased hearts [76,78-80]. TIMPs can inhibit several MMPs, while they each also possess unique properties [81]. Although MMPs and TIMPs are best known for their functions in ECM homeostasis, they also possess a number of other functions and properties that have been discussed elsewhere [64,82].

CFBs can produce a number of MMPs and TIMPs [83-86] whereby they can impact different aspects of ECM homeostasis and remodeling. A number of growth factors, cytokines, and chemokines have been identified that can regulate production of MMPs and TIMPs by CFBs. Proinflammatory cytokines such as TNFα and IL-1β induce transcription of a number of MMPs, TIMP1 and TIMP2 in the myocardium [87]. Brain natriuretic peptide (BNP) has been reported to be produced by CFBs and to induce production of MMP1, MMP2, MMP3, MMP14 and TIMP2 [88]. Adult mouse FBs have been reported to synthesize a number of soluble secreted MMPs (MMP13, MMP8, MMP2, and MMP9), and two MT-MMPs (MMP14 (MT1-MMP) and MMP16 (MT3-MMP)) [83]. Using a range of MMP-deficient mice (MMP13^−/−^, MMP8^−/−^, MMP2^−/−^, MMP9^−/−^, MMP14^−/−^ (or MT1-MMP^−/−^) and MMP16^−/−^ (or MT3-MMP^−/−^)), Sabeh and colleagues demonstrated that only the membrane-anchored MMP14 is required for focal collagen invasion required for FB migration through the stroma, compared with bulk collagenolysis by the soluble MMPs [89].

While CFBs are the main source of ECM regulatory proteins, MMPs and TIMPs, these molecules can also impact on CFB function. MT1-MMP can cleave a number of ECM proteins including fibronectin, laminin-1 and fibrillar collagen type I [70-73], and has been shown to also trigger fibrosis by cleaving and activating the latent ECM-bound TGFβ, activating the Smad pathway in CFBs and triggering collagen production [90,91]. MMP2 and MMP9 have been shown to release the ECM-bound latent TGFβ, thereby inducing collagen synthesis [92]. Consistently, cardiac overexpression of MMP2 led to severe myocardial fibrosis [93]. In quiescently cultured human CFBs, overexpression of TIMPs using specific adenoviruses showed that each TIMP can impact the function of CFB differently [94]. Overexpression of Ad-TIMP1, Ad-TIMP2, Ad-TIMP3 and Ad-TIMP4 increased αSMA levels, indicating differentiation of CFBs into myoFBs. Ad-TIMP2 increased collagen synthesis by CFBs, whereas Ad-TIMP3 increased FB apoptosis. These functions of TIMPs were independent from their MMP-inhibitory function [94]. These findings collectively indicate that while CFBs produce ECM proteins and the ECM-regulatory proteins, they are in turn influenced by these factors working as a self-regulating cycle.

### Remodeling of myocardial extracellular matrix in heart disease patients

Remodeling of the ECM is a key component of cardiac remodeling that occurs in disease. Disruption of the ECM network structure interrupts the connection between the myocardial cells and blood vessels, thereby compromising the structural integrity and function of the heart. On the other hand, excess production and accumulation of ECM structural proteins, or fibrosis, results in enhanced stiffness of the myocardium and impedes ventricular contraction and relaxation, leading to distorted architecture and function of the heart. Excess collagen deposition and fibrosis has been clearly linked to myocardial stiffness, diastolic and systolic dysfunction [95]. Fibrosis can be the result of hyperactivity of existing FBs that proliferate rapidly in response to injury, or recruitment and proliferation of circulating bone-marrow-derived cells that can enter the myocardium and transform into FBs and myoFBs.

**Figure 3:**
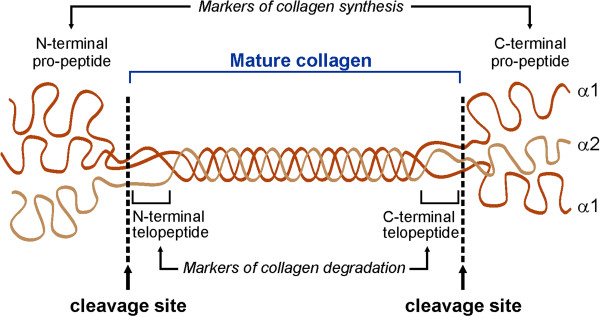
**Structure of collagen molecule.** Pro-collagen is comprised of two alpha-1 chains and one alpha-2 chain intertwined into a triple helix. Pro-peptide domains at the carboxy-terminals and amino-terminals are cleaved, resulting in formation of mature collagen. When collagen is degraded, during physiological turnover or pathological adverse remodeling, telopeptides (from the amino-terminals or carboxy-terminals) are cleaved and released into the plasma.

Fibrillar collagen types I and III are the predominant components of cardiac ECM. These collagens are produced as pro-collagens that are then processed into mature collagen molecules upon cleavage of their pro-peptide domain by procollagen peptidase. Assembly and cross-linking of mature collagen molecules gives rise to collagen fibrils and collagen fibers. During physiological ECM turnover or pathological ECM remodeling, collagen fibers are degraded and the telopeptides in the amino-terminals or carboxy-terminals of collagen molecules are cleaved (Figure 3). The pro-peptide from the carboxy-terminal or the amino-terminal propeptides of collagen type I (PICP, PINP), and those of collagen type III (PIIICP, PIIINP) are released during biosynthesis of these collagens in a stoichiometric manner, and hence are considered biomarkers of collagen synthesis. However, the carboxy-terminal or amino-terminal telopeptide of collagen type I (CITP, NITP) and type III (CIIITP, NIIITP), which are produced when these collagens are degraded, are considered biomarkers of collagen degradation [96]. Measurement of these biomarkers in heart disease patients has provided insight into cardiac ECM remodeling in different types of heart disease.

Different reports have been made with respect to collagen turnover in hypertensive patients. Reduced serum CITP, a marker of collagen type I degradation, was suggested to explain collagen deposition and fibrosis in hypertensive patients [97], whereas a later study showed increased CITP as well as PICP (a marker of collagen synthesis) in hypertensive patients with left ventricular (LV) fibrosis and diastolic dysfunction indicating increased overall collagen turnover in these patients [98]. Using endomyocardial biopsies from hypertensive patients, a direct correlation was found between serum PICP and collagen volume fraction, while PICP levels were also higher in patients with severe fibrosis compared with those with less severe fibrosis [99]. Similarly, serum levels of PINP correlated with diastolic dysfunction in hypertensive patients without diabetes [100], suggesting that a serum marker of collagen synthesis (PICP or PINP) could be used as a biomarker for fibrosis in hypertensive patients. In hypertrophic cardiomyopathy, ECM turnover is a major determinant of cardiac remodeling. In patients with congestive heart failure, high serum levels of cardiac fibrosis (PIIINP and PICP) are significantly associated with poor outcome [101].

In patients with coronary artery disease but no MI, serum levels of PIIINP, but not PINP, correlated well with the number of diseased vessels and severity of coronary artery disease [102]. In addition, in patients with acute myocardial infarction (AMI), elevated serum PIIINP levels during the first few days post MI was associated with suppressed LV function, increased LV volume over 1-year follow-up [103], poor overall prognosis and survival [104]. Early post-MI serum PIIINP levels have been suggested to serve as a marker of long-term LV remodeling and prognosis. However, a more recent study reported that CITP served as the most suitable prognostic tool in patients with acute and chronic MI compared with PINP, PIIINP and TIMP1 [105].

In using plasma biomarkers for ECM remodeling in patients with heart disease, it is important to keep in mind that collagen type I is the most abundant collagen in the human body and is ubiquitously expressed in almost all organs and tissues in the body. Although collagen type III shows a relatively more tissue-specific expression pattern, it is also highly expressed in the skin, lungs and the vasculature. Hence, development of more cardiac-specific plasma biomarkers would provide more accurate insight into ECM alterations in the myocardium.

### Alterations in MMPs and TIMPs in heart disease patients

Alterations in MMPs and TIMPs levels have been studied in different types of heart disease as a surrogate measure of myocardial ECM remodeling. In patients with end-stage dilated cardiomyopathy, analysis of LV myocardial tissue showed decreased MMP1, increased MMP3, MMP9, TIMP1 and TIMP2, and no changes in MMP2 levels [106]. Picard and colleagues reported increased MMP1 and TIMP1 mRNA levels in right septal endomyocardial biopsies from patients with dilated cardiomyopathy that did not correlate with LV diameter, whereas collagen volume density correlated well with LV diameter in these patients [107]. Hypertensive patients with cardiac hypertrophy have been reported to have reduced plasma levels of MMP1 [97], MMP2 and MMP9 [108], while elevated plasma TIMP1 levels have been reported in hypertensive patients [97] that correlated with diastolic dysfunction and LV fibrosis [98].

In patients with MI and unstable angina, serum levels of MMP2 and MMP9 (but not MMP1), TIMP1, TNFα and IL-6 were significantly elevated compared with healthy controls, suggesting that these MMPs, TIMP1 and proinflammatory cytokines could play an important role in the pathophysiology of acute coronary syndrome [109]. Measurement of temporal changes in plasma MMPs and TIMPs levels following MI showed a rapid and sustained increase in MMP9 and MMP8, with a delayed increase in TIMP2 and TIMP4 levels [110]. Plasma levels of MMP2 and MMP9 were elevated in AMI patients, but only the MMP9 levels exhibited a biphasic profile that peaked within the first 12 hours and then fell to a plateau [110]. This report is consistent with an earlier study that reported the early peak in MMP9 levels correlated with white blood cell and neutrophil counts after AMI, and inversely correlated with LV ejection fraction and LV end-diastolic volume during the follow-up, whereas the higher plateau levels later after AMI were associated with better LV function and LV remodeling [111]. However, plasma MMP9 has also been reported to serve as a useful prognostic tool in patients with AMI, where during the 2-year follow-up plasma MMP9 levels (but not MMP2, TNFα, C-reactive protein, creatine kinase or pro-BNP) were the only predictive of late-onset congestive heart failure [112]. Overall, plasma biomarkers and their levels can be influenced by the type, severity and stage of disease, which should be taken into consideration when comparing reports from different studies.

### Experimental models of heart disease and extracellular matrix remodeling

MI results from occlusion of a coronary artery, subjecting the downstream myocardial tissue to hypoxia and ischemia. The remodeling process consists of a series of timed molecular events that include recruitment of inflammatory cells, proliferation of CFBs or recruitment of circulating bone-marrow-derived cells and their differentiation to myoFBs, and formation of the fibrotic scar tissue. Experimental models of myocardial ischemic injury can be generated in different species by permanent or temporary ligation of the left anterior descending coronary artery resulting in MI or ischemia–reperfusion, respectively. Ischemia–reperfusion results in greater inflammatory cell influx and prolonged inflammatory response compared with MI, whereas in the MI model a greater number of CFBs are detected at the injury site, which correlates well with greater degree of fibrosis and ECM remodeling in the MI compared with the ischemia–reperfusion model [113]. CFBs are a critical element of myocardial repair that produce collagens, providing the tensile strength for cardiac tissue [1]. As such, interruption or hindrance of CFB activation will lead to decreased tensile strength of the cardiac wall, predisposing the cardiac chambers to dilate from the pressure of the blood within the chamber. Although inhibition of fibrosis post MI in mice lacking secreted frizzled-related proteins-2 was reported to result in beneficial outcomes [114], a recent study showed that inhibiting CFBs (by interrupting the wnt/β-catenin signaling) prevented fibrosis, impaired the wound healing, and accelerated cardiac dilation and dysfunction within a few days of myocardial ischemic injury in mice [115]. This study clearly indicates that the post-MI fibrosis is reparative, and in fact a healing process and interruption of this process may bear unfavorable outcomes [116]. The wnt-1/β-catenin has also been identified as a key pro-fibrotic signaling pathway in myocardial ischemia–reperfusion injury, activating the epicardial cells to undergo epithelial-to-mesenchymal transformation, generating FBs, triggering CFB proliferation and expression of profibrotic genes [116]. Following myocardial ischemic injury, MMPs mediate a number of cellular responses, such as inflammation and fibrosis, through processing the ECM proteins as well as non-ECM substrates. MMP-mediated degradation of the ECM generates fragments that serve as chemoattractants triggering infiltration of inflammatory cells to the site of injury. The infiltrating macrophages and neutrophils in turn produce a number of MMPs, such as MMP8, MMP9 and MMP12 [117-120], which then further contribute to the adverse remodeling.

In hypertensive heart disease, reactive myocardial fibrosis increases myocardial stiffness and reduces compliance. Roles of MMPs and TIMPs have been indicated in the myocardial fibrosis in a number of hypertensive animal models. Deoxycorticosterone acetate salt-hypertensive rats showed higher collagen deposition through endothelin-1-mediated TGFβ expression [121]. This was later reported to be preceded by increased fibronectin expression, which could contribute to ECM cell attachment and promote collagen deposition, as well as elevated gelatinase levels (MMP2 and MMP9) [122].

While TGFβ is well known to activate the Smad signaling pathway in CFBs, thereby mediating FB activation and collagen production, a recent study by Koitabashi and colleagues demonstrated that the TGFβ signaling pathway in the cardiomyocytes via TGFβ-receptor 2 plays a critical role in myocardial fibrosis following pressure overload [123]. Cardiomyocyte-specific knockdown of TGFβ-receptor 2 completely blocked myocardial fibrosis and LV dysfunction, activation of the Smad pathway as well as TGFβ-activated kinase 1 and preserved capillary density. However, cardiomyocyte knockdown of TGFβ-receptor 1 or treatment with a TGFβ neutralizing antibody only suppressed the Smad activity (not TGFβ-activated kinase 1) and partially suppressed fibrosis without improving LV function [123]. As such, TGFβ-mediated activation of TGFβ-activated kinase 1in cardiomyocytes was proposed to underlie the maladaptive hypertrophy and dysfunction secondary to cardiac pressure overload.

Atrial natriuretic peptide and BNP have been shown to inhibit FB proliferation, collagen synthesis and MMP release via activation of the cGMP pathway [124], and to oppose the TGFβ-induced ECM protein synthesis *in vitro *[125,126]. These findings are particularly interesting since FBs are also an important source of natriuretic peptides, and as such can generate a negative feedback loop [127]. Deletion of the major natriuretic receptor for atrial natriuretic peptide and BNP, natriuretic peptide receptor-1 in mice (Npr1^−/−^), resulted in hypertension, cardiac hypertrophy, congestive heart failure and sudden death at 6 months of age [128,129]. Elevated levels of MMP2, MMP9 and TNFα in these mice were linked to increased production of collagen types I and III by CFBs in a TGFβ-dependent manner, leading to myocardial fibrosis [130]. Atrial natriuretic peptide and BNP levels are consistently elevated in heart disease in patients [131,132] as well as in experimental models of heart disease [133,134], and are consistently linked to severity and progression of disease [135]. Taken together, these elevated atrial natriuretic peptide and BNP levels in heart disease could be a protective attempt by the myocardial tissue to limit excess fibrotic deposition, tissue injury and adverse remodeling.

### MMPs, TIMPs, myocardial remodeling and fibrosis

As discussed earlier, a tightly controlled balance between the function of MMPs and TIMPs is critical in maintaining the ECM integrity. A number of experimental animal models have been developed in order to determine the contribution of MMPs and TIMPs in ECM remodeling in heart disease, and genetically modified mice have provided valuable tools in examining the causal role of MMPs and TIMPs in this process.

#### Remodeling following myocardial infarction

Targeted deletion of MMP2 improved post-MI survival by hindering macrophage infiltration and reducing the rate of LV rupture [136]. MMP7 has been shown to cleave the gap junction connexin-43, thereby promoting an arrythmogenic response post MI. MMP7 deletion therefore improved post-MI survival and improved myocardial conduction pattern owing to preserved connexin-43 levels [137]. MMP9 deletion provided partial protection against post-MI rupture [138], while reducing LV dilation and dysfunction [139]. Lack of individual TIMPs influenced different aspects of cardiac structure and function following MI. In TIMP1^−/−^ mice, MI led to greater LV dilation and increased LV end-diastolic volume compared with parallel wildtype mice [140]. In mice lacking TIMP2, MI exacerbated LV dilation and reduced the ejection fraction but did not alter the rate of LV rupture compared with WT-MI mice [78]. Lack of TIMP3 increased the rate of LV rupture, worsened LV dilation and reduced ejection fraction following MI [133], whereas TIMP4 deficiency only increased the rate of LV rupture without affecting the LV structure or function post MI [141]. While lack of TIMP2 and TIMP3 exacerbated infarct expansion, the increased rate of LV rupture in TIMP3^−/−^ and TIMP4^−/−^ impaired ECM remodeling in these mice. Second harmonic generation imaging further revealed reduced density and greater disarray of fibrillar collagens in the infarct myocardium of TIMP3^−/−^ and TIMP4^−/−^, consistent with the increased rate of LV rupture in these mice [133,141]. These data indicate that while TIMP1, TIMP2 and TIMP3 exert a global impact altering the overall structure and function of the LV myocardium, the function of TIMP4 appears to be localized to the infarcted myocardium.

Mice overexpressing MT1-MMP showed lower survival and ejection fraction post MI compared with parallel wildtype mice, whereas these parameters were improved in mice with reduced MT1-MMP levels (MT1-MMP^+/−^) [90]. Overexpression of TIMP1 has been shown to have beneficial effects in mouse [142] and rat [143] models of MI. Additionally, overexpression of TIMP2 in the peri-infarct myocardium reduced the infarct expansion and improved LV dilation and dysfunction [144].

#### Fibrosis and ECM remodeling in hypertension and cardiac pressure overload

Pressure overload exerts a mechanical stress on the ventricles and can trigger cardiac hypertrophy and fibrosis. In this model of heart disease, the excessive biomechanical stress is transmitted to ECM and cell–ECM connections that can lead to adverse remodeling of the ECM, and can further activate the intracellular signaling pathways leading to cardiac hypertrophy, fibrosis and cell death. MMP2-deficient mice showed reduced myocardial hypertrophy and fibrosis [145], while MMP9 deficiency partially improved myocardial hypertrophy and fibrosis following pressure overload [146]. We recently reported that in response to cardiac pressure overload, TIMP2^−/−^ mice exhibit greater LV dilation and dysfunction, with non-homogeneous ECM remodeling which was characterized by areas of disrupted ECM network adjacent to regions of fibrotic lesions [134]. Myocardial fibrosis in pressure-overloaded TIMP2^−/−^ hearts was not due to increased expression of collagen type I and/or type III, however, but due to elevated levels of SPARC (secreted protein acidic and rich in cysteine) and enhanced post-translational stabilization of collagen fibers [134]. Cardiac pressure overload in TIMP3-deficient mice led to exacerbated LV remodeling, and dysfunction [147], and to severe myocardial fibrosis [148]. The exacerbated LV dilation and dysfunction in these mice was found to be due to the combined contribution of augmented MMP-mediated proteolytic activities and heightened the TNFα-converting enzyme–TNFα pathway [147], while myocardial fibrosis was found to be mediated through an interaction between the TNFα and TGFβ pathways that led to increased expression of fibrillar collagens [148]. Interestingly, TIMP4 was found not to contribute to cardiac response to mechanical stress, as TIMP4^−/−^ mice exhibited comparable cardiac remodeling, dysfunction and myocardial fibrosis compared with the parallel wildtype mice [141].

## Conclusion

The ECM is an integral component of the myocardium, and the factors that influence the integrity of the ECM structure also impact cardiac structure and function. Cardiac FBs play a central role in the physiological turnover of the ECM as well as its pathological remodeling. Although cardiac FBs are often associated with cardiac fibrosis and adverse outcomes, it is important to note that the primary function of FBs is tissue repair (wound healing) – which in cases such as MI is in fact beneficial, and its interruption would have undesirable outcomes. In addition, MMPs are traditionally known for degrading the ECM proteins, and TIMPs to inhibit this process. However, MMPs can also promote ECM production (and fibrosis) by regulating the activity of FBs, and similarly TIMPs can influence FB behavior and ECM production in a MMP-independent fashion. Hence, it is critical to understand the diverse functions of MMPs, TIMPs and FBs towards developing effective therapies to control harmful myocardial fibrosis.

## Abbreviations

AMI, acute myocardial infarction; αSMA, alpha smooth muscle actin; BNP, brain natriuretic peptide; CFB, cardiac fibroblast; CITP, carboxy-terminal telopeptide of collagen type I; CIIITP, carboxy-terminal telopeptide of collagen type III; DDR, discoidin domain receptor; ECM, extracellular matrix; FB, fibroblast; IL, interleukin; LV, left ventricular; MI, myocardial infarction; miRNA, microRNA; MMP, matrix metalloproteinase; MT-MMP, membrane-type matrix metalloproteinase; myoFB, myofibroblast; NITP, amino-terminal telopeptide of collagen type I; NIIITP, amino-terminal telopeptide of collagen type III; PICP, carboxy-terminal propeptides of collagen type I (or procollagen type I carboxy-terminal propeptide); PINP, amino-terminal propeptides of collagen type I (or procollagen type I amino-terminal propeptide); PIIICP, carboxy-terminal propeptides of collagen type III (or procollagen type III carboxy-terminal propeptide); PIIINP, amino-terminal propeptides of collagen type III (or Procollagen type III amino-terminal propeptide); TIMP, tissue inhibitor of metalloproteinase; TGFβ, transforming growth factor beta; TNF, tumor necrosis factor.

## Competing interests

The authors declare that they have no competing interests.

## Authors’ contributions

DF and ZK conceived the original concept of the review. DF contributed to 50%, and AT and JL each contributed to 25% of the first draft. All authors read and approved the final manuscript.
